# Analysis of Patient Education Guides Generated by ChatGPT and Gemini on Common Anti-diabetic Drugs: A Cross-Sectional Study

**DOI:** 10.7759/cureus.81156

**Published:** 2025-03-25

**Authors:** Jyothis G Saji, Aswini Balagangatharan, Somya Bajaj, Vibha Swarnkar, Devika Unni, Aswathy Dileep

**Affiliations:** 1 Emergency Medicine, Jubilee Mission Medical College & Research Institute, Thrissur, IND; 2 Cardiology, Dr. Madhavan's Heart Centre, Madurai, IND; 3 Internal Medicine, Sir Seewoosagur Ramgoolam Medical College, University of Mauritius, Belle Rive, MUS; 4 Internal Medicine, Eureka Hospital and Research Center, Indore, IND; 5 Nephrology, VPS Lakeshore Hospital, Kochi, IND; 6 Internal Medicine, Malabar Medical College, Modakkallur, IND

**Keywords:** artificial intelligence, chatgpt, google gemini, insulin, metformin

## Abstract

Introduction

Patient information guides are required, as they help to identify early diseases and complications and to prevent them. Artificial intelligence (AI) tools are being used to create patient education guides for easy accessibility to people.

Methodology

This was a cross-sectional study analyzing patient information guides regarding six anti-diabetic drugs created using ChatGPT (OpenAI, Inc., San Francisco, California, United States) and Gemini (Google LLC, Mountain View, California, United States), respectively. The patient information guides were on anti-diabetic drugs, including metformin, empagliflozin, liraglutide, glipizide, sitagliptin, and insulin glargine, and were created with the help of various prompts.

Results

There was no statistically significant difference found between any of the characteristics of the responses generated by the two AI tools, according to the P values obtained.

Conclusion

On comparing the two AI tools, there was not much difference noted in readability, reliability, and similarity.

## Introduction

Anti-diabetic drugs are medications that control blood glucose levels in diabetic patients by enhancing insulin secretion or sensitivity or decreasing glucose production and absorption [1]. Patient education is crucial since it prevents complications, early mortality, and morbidity by assisting with early problem detection, medication management, etc. [2]. 

Artificial intelligence (AI) tools are computer programs that use algorithms to analyze data, automate processes, and support decision-making in a variety of fields. These can be beneficial for patient education in the development of patient guides providing simple medical information that helps patients to easily understand their medical conditions, treatments, and management care [3]. However, there are a few drawbacks to using AI tools in patient education, like potential misinformation, lack of personalization, privacy concerns, and overreliance on technology, which can hinder effective decision-making for patients [4]. 

ChatGPT (OpenAI, Inc., San Francisco, California, United States) and Gemini (Google LLC, Mountain View, California, United States) are advanced AI language models that are built on different architectures and trained differently. Gemini from Google has a retrieval-augmented generation (RAG) system bringing in outside search results to ensure facts and supports multimodal (text, images) input. It is very good at tasks that are based on fact. On the other hand, OpenAI's ChatGPT has been specifically optimized for conversational fluency and creative tasks, building on reinforcement learning with human feedback (RLHF) to strengthen dialogue. Gemini is geared toward precision and real-world problem-solving, while ChatGPT excels in creative, open-ended conversations [5].

Healthcare professionals would benefit from AI tools through access to personalized patient education resources tailored to reading levels and assistance in clinical decision-making through real-time data analysis as well as workload reduction via virtual health assistants managing routine patient inquiries and medication reminders [6]. Deployment of AI systems in healthcare environments should be focused on humans' health and well-being while adhering to bioethical standards such as autonomy, nonmaleficence, beneficence, and justice. Robust AI governance should ensure definitive accountability while undertaking comprehensive risk assessments to ensure both transparency and security of the technology as well as the protection of patient rights [7]. 

The role of these two advanced AI tools, ChatGPT and Gemini, in patient counseling on anti-diabetic drugs is significant as they provide easily accessible information about medication options, dosage, side effects, and lifestyle modifications to the patients and their caregivers [8]. The aim and objective of this study were to compare responses by ChatGPT and Gemini related to patient education guides on common anti-diabetic drugs like metformin, empagliflozin, liraglutide, glipizide, sitagliptin, and insulin glargine based on readability, reliability, and similarity percentage.

## Materials and methods

This was a cross-sectional original research study to assess the readability and reliability of patient education brochures generated by ChatGPT and Gemini based on six common anti-diabetic drugs: metformin, empagliflozin, liraglutide, glipizide, sitagliptin, and insulin glargine. The study had a duration of one week (October 13-19, 2024) and was solely based on data generated by two AI tools: ChatGPT (version 4.0) [9] and Gemini (version 1.5) [10]. No ethics approval was necessary since the study did not include human subjects or sensitive data. All data collection and analysis were done virtually, using digital tools and software.

ChatGPT and Gemini were chosen because they are widely used and are two of the most sophisticated AI systems available for generating patient educational material. Both the tools were asked to produce patient education guides for the six common anti-diabetic drugs in their default and standard settings, without any fine-tuning or modifications, using the same prompts to ensure consistency in the inputs: “Write a patient education guide for metformin”, “Write a patient education guide for empagliflozin”, “Write a patient education guide for liraglutide”, “Write a patient education guide for glipizide”, “Write a patient education guide for sitagliptin” and “Write a patient education guide for insulin-glargine”. 

The study utilized AI-generated patient education guide content based solely on the initial prompt without further prompts to simplify the output. Both tool outputs were formatted in a Microsoft Word file (Microsoft Corporation, Redmond, Washington, United States) without any adjustment or modifications to preserve the initial AI-generated content. The outputs were subsequently assessed with standardized tools, including the Flesch-Kincaid Calculator for readability assessment [11], the QuillBot Plagiarism Tool for content similarity assessment [12], and the modified DISCERN Score for reliability assessment as in the study by Uzun [13].

Measurement instruments

The primary outcome measures were the readability and reliability of AI-generated brochures. Using the Flesch-Kincaid calculator, which calculates the Flesch Reading Ease Score (FRES) and Flesch-Kincaid Grade Level (FRGL), quantified readability according to the following metrics: word count (total number of words in the text), sentence count (total number of sentences), average words per sentence (measures sentence complexity), average syllables per word (indicates word complexity), grade level (an estimate of the education level needed to comprehend the text), and ease score (via a 0-100 scale, with higher numbers indicating better readability) [11]. 

The AI content uniqueness was measured by calculating the similarity percentage using the QuillBot Plagiarism Tool [12]. This was used to assess whether the AI tools were actually creating original content or simply copying from other published sources.

The modified DISCERN score was used for reliability assessment [13]. The modified DISCERN scale uses five questions created from the DISCERN tool used to evaluate the credibility of health information. In order to obtain a score according to the modified DISCERN scale, each question scored 0 or 1. In this rating system, a high score means great reliability (5), and the lower the score, the lower the reliability (0).

Data analysis

Data were organized in a Microsoft Excel sheet (Microsoft Corporation) for further analysis. The data were analyzed using R version 3.2.4 (R Foundation for Statistical Computing, Vienna, Austria). A comparative analysis of the mean value of the word count, sentence count, average words per sentence, average syllables per word, grade level, ease score, similarity %, and reliability score of ChatGPT and Gemini was performed using an unpaired t-test. Statistical significance was set at p-value < 0.05.

## Results

The characteristics of the responses produced by Gemini and ChatGPT are displayed in Table 1. The brochures produced by ChatGPT were found to have a greater mean word count (n = 422.83) and more sentences (n = 51.50) than those produced by Gemini, with a mean word count of 404.67 and 42 sentences. In all the domains examined, including word count, sentence count, average syllables per word, grade level, ease score, similarity percentage, and reliability score, there were no statistically significant differences.

**Table 1:** Characteristics of the responses produced by Gemini (Google LLC) and ChatGPT (OpenAI Inc.) t-test; P-values <0.05 are considered statistically significant.

Parameters	ChatGPT		Gemini		P value
	Mean	Standard Deviation	Mean	Standard Deviation	
Words	422.83	140.70	404.67	133.11	0.823
Sentences	51.50	14.24	42.00	13.21	0.258
Average Words per Sentence	8.25	1.56	9.77	1.61	0.129
Average Syllables per Word	1.87	0.05	1.83	0.05	0.290
Grade Level	9.65	0.65	9.85	0.67	0.609
Ease Score	40.53	3.92	41.83	3.95	0.580
Similarity Percentage	28.45	12.36	32.15	11.80	0.607
Reliability Score	3.50	0.55	3.50	0.55	1.000

**Figure 1:**
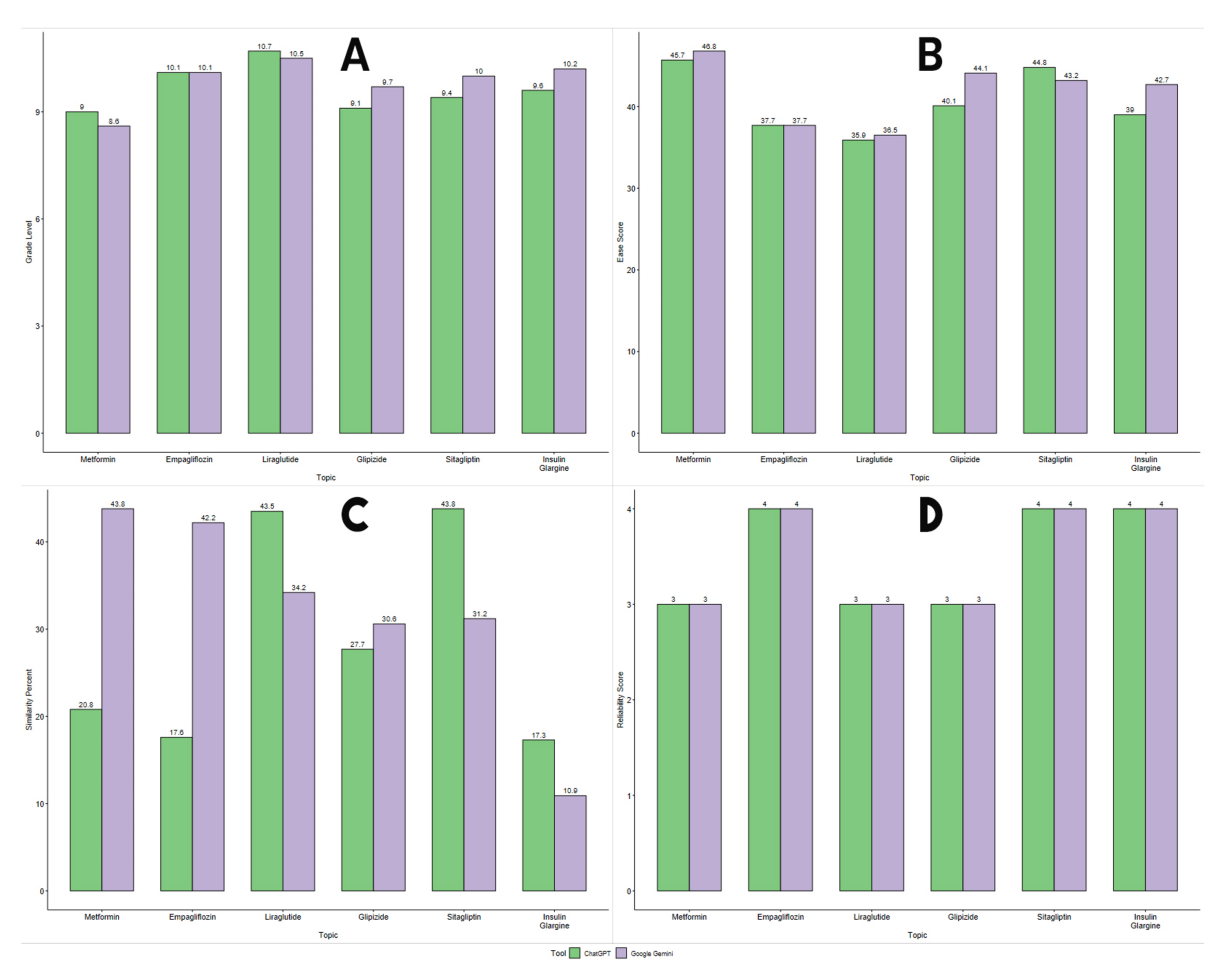
Comparison of the grade level (A), ease score (B), similarity percentage (C), and reliability score (D) for the patient education guides produced by ChatGPT (OpenAI Inc.) and Gemini (Google LLC)

A comparison of the grade level, ease score, similarity percentage, and reliability score for the patient education guides produced by ChatGPT and Gemini is shown graphically in Figure 1. Based on the grade level comparison, the results of Gemini and ChatGPT were almost identical (Figure 1A). Out of the six anti-diabetic drugs, Gemini had a higher grade level in three anti-diabetic drugs (glipizide: 9.7, sitagliptin: 10, and insulin glargine: 10.2), whereas ChatGPT had a higher grade level in two anti-diabetic drugs (metformin: 9, liraglutide: 10.7), and the grade level was found to be equal for one anti-diabetic drug (empagliflozin: 10.1).

While assessing the ease score (Figure 1B), Gemini's guide scored slightly greater in four drugs (metformin, liraglutide, glipizide, and insulin glargine) than ChatGPT's, with a pronounced difference for glipizide (ease score of ChatGPT=40.1, ease score of Gemini=44.1) and insulin glargine (ease score of ChatGPT=39, ease score of Gemini=42.7), while the ease score for ChatGPT was slightly greater in sitagliptin (ease score of ChatGPT=44.8, ease score of Gemini=43.2) and was found to be equal in the case of empagliflozin (37.7). This suggested that the text produced by Gemini was simpler to read.

When the similarity percentages of the educational guide were compared (Figure 1C), it was found that ChatGPT had a significantly higher percentage of similarity on liraglutide (43.5%), sitagliptin (43.8%), and insulin glargine (17.3%), whereas Gemini had a higher percentage of similarity on metformin (43.8%), empagliflozin (42.2%), and glipizide (30.6%). The patient educational guides created by both ChatGPT and Gemini on all anti-diabetic medicines had a similar reliability score (Figure 1D).

## Discussion

This cross-sectional study comparing two AI tools, ChatGPT and Gemini, for patient education brochures on metformin, empagliflozin, liraglutide, glipizide, sitagliptin, and insulin glargine observed no statistically significant difference. It was observed that ChatGPT had a grade level of 9.65 and Gemini of 9.85 (p value=0.609). ChatGPT has an ease score of 40.53, compared to Gemini's 41.83 (p-value=0.580). The similarity percentages for ChatGPT and Gemini were 28.45 and 32.15, respectively (p-value=0.607). Lastly, it was discovered that the reliability score for ChatGPT and Gemini was 3.50 (p-value = 1.000).

Personalized content helps develop a deeper understanding of medical information by AI in educating the patient. Such AI tools, such as chatbots and virtual assistants, can make teaching-learning environments become more engaging and accessible, from simplifying health topics to answering patient inquiries in real time [14]. Many online medical articles are not readable or understandable for the common people or non-medical professionals. AI has helped condense and simplify information from all over the internet. This study aimed to assess the ease of reading brochures created by ChatGPT and Gemini. The difficulty level of this information was determined through the FRES. To read an article, a minimum Flesch score of 30-50 is required for college students [15]. The study discovered an ease score of 40.53 for ChatGPT and 41.83 for Gemini, indicating that both AI articles could be read by a college student. A brochure is considered easy to read if its ease score is high enough (50-60) to be easily comprehensible by a high school student. However, neither ChatGPT nor Gemini had such a score. This differs from the study by Adithya et al. (2024), which reported that Gemini had a Flesch-Kincaid grade level of 50.62, indicating that it is readily readable and comprehended by high school children [16].

The AI models may produce precisely similar or identical words since they are mostly trained on an immense dataset of previously published information, thus raising alarms regarding accidental plagiarism. Medical plagiarism becomes a dangerous affair as it undermines the scientific integrity of the work and reduces confidence in published research by propagating unreliable or misrepresented material, which can affect patient care and clinical decision-making. The results of the current study showed that the similarity percentage was higher for Gemini than ChatGPT. According to a study by Sallam (2023), problems concerning ChatGPT use were identified in 58 out of 60 (96.7%) records. These concerns included issues with ethics, copyright, transparency, lack of originality, plagiarism, bias, and inaccurate content with the potential for hallucinations, limited knowledge, the risk of infodemics, cybersecurity, and incorrect citations [17].

The modified DISCERN score is a revised DISCERN tool that has now been adjusted for evaluating the quality of written health information. It also assesses online health content in terms of clarity, relevance, and evidence-based accuracy. Higher scores on a scale of 1 to 5 indicate better quality. According to this study, the average DISCERN score for Gemini and ChatGPT was 3.5, suggesting moderate quality that might be improved. This finding is comparable to a study by Behers et al. (2024), which found that ChatGPT delivered the most reliable and high-quality materials, followed closely by Gemini, among other AI models such as Meta AI and Microsoft Copilot in educating patients on cardiac catheterization [18].

One of the most serious issues is that AI tools do not check the quality of the scientific references they use to generate the information, which may affect the accuracy of results. Additionally, the chatbots are not updated often, so they can generate results from outdated studies. Another drawback is the difficulty in accessing paid/subscription medical articles, meaning the AI tool might not always deliver the most recent medical scientific data and may extract information only from openly available or open-access articles [19].

Limitations of the study

This study only used two AI tools; additional AI tools may have been evaluated to produce better findings. For more clarity, more drugs may have been added. The study also did not analyze patient-specific health factors like comorbidities and medical histories, which may impact medication effects, nor did it assess AI systems' ability to integrate cultural sensitivities that contribute to patient adherence and understanding. Patients' assessments should have been included in the analysis.

Our study was restricted to English-language content, which prevented us from examining translation quality issues, medical terminology differences, and cultural adaptation challenges in non-English materials, thus limiting the worldwide relevance of our results. The tools also have the potential to generate information that is both inaccurate and biased, which can negatively affect patient safety and comprehension. However, this study did not include a detailed fact-checking process, and the accuracy of the generated facts was not verified, which is a limitation, as inaccuracies or biases in the generated content could impact the reliability and effectiveness of patient education materials. Furthermore, while this study determined the readability of AI-written content, it is important to clarify that readability is distinct from comprehension. The words may seem simple in many medical documents, but patients can still find it difficult to comprehend or remember the medical content.

A major limitation was that the results from AI tools demonstrated significant variability. Content generated by ChatGPT (version 4.0) and Gemini (version 1.5) can vary because algorithm updates or prompt modifications affect result consistency and content comparability. The reliability evaluation lacked transparency because scoring ambiguous answers using the modified DISCERN score relied on undefined criteria, which could lead to subjective evaluation results. The inter-rater reliability for the modified DISCERN scores was also not directly addressed, as there was no specific information on how to manage responses falling into the ambiguous or borderline category, introducing subjectivity into the reliability assessments.

## Conclusions

This study aimed to compare two AI tools in generating responses for writing patient education guides on common anti-diabetic drugs based on readability, ease of understanding, reliability, and similarity. The results showed that there was not much difference in the responses generated by both AI tools in terms of word count, sentence count, average words per sentence, average syllables per word, grade level, ease score, reliability score, and similarity percentage. However, this study only focused on two AI platforms, and researchers need to study more AI platforms to improve the efficiency and generalizability of future studies. As this study utilized ChatGPT version 4.0 and Gemini version 1.5 specifically, it may not be generalizable to all AI models or versions, and the research scope must be broader.

AI-generated materials often fall short of ideal patient education standards, as they face significant challenges in factual accuracy, producing inconsistent, inaccurate, or false information that can confuse or mislead patients, which is particularly concerning in medical education. AI-based content generation tools cannot serve as the exclusive source for medical education because human oversight is necessary to maintain accuracy and alignment with current medical practices. Future research should focus on improving AI tool reliability and working on proper assessment guidelines to spread the use of AI in medical education. Resolving these concerns is important to ensuring that AI-produced medical content adheres to ethical standards.
